# Inhibition of Key Citrus Postharvest Fungal Strains by Plant Extracts In Vitro and In Vivo: A Review

**DOI:** 10.3390/plants8020026

**Published:** 2019-01-22

**Authors:** Jinyin Chen, Yuting Shen, Chuying Chen, Chunpeng Wan

**Affiliations:** 1 Jiangxi Key Laboratory for Postharvest Technology and Nondestructive Testing of Fruits & Vegetables, Collaborative Innovation Center of Postharvest Key Technology and Quality Safety of Fruits and Vegetables, Jiangxi Agricultural University, Nanchang 330045, China; jinyinchen@126.com (J.C.); ytshenchina@126.com (Y.S.); cy.chen@jxau.edu.cn (C.C.); 2 Pingxiang University, Pingxiang 337055, China

**Keywords:** plant-derived fungicides, *Penicillium digitatum*, *Penicillium italicum*, citrus preservation

## Abstract

*Citrus* fruits are subjected to a diversity of postharvest diseases caused by various pathogens during picking, packing, storage and transportation. Green and blue molds, caused by *Penicillium digitatum* and *Penicillium italicum*, respectively, are two major postharvest citrus diseases and cause significant economic losses during the commercialization phase. Currently, the control of postharvest citrus diseases relies mainly on the use of synthetic fungicides, which usually result in the resistance against fungal attack, environment pollution and health hazards. In recent years, much attention has been given to the preservation of citrus by naturally isolated edible plant extracts, medicinal plant extracts, *Citrus* extracts and volatiles, et al. Scientists worldwide devote their time and energy to discover the high effect, low toxicity, safety and inexpensive plant-derived fungicides. The current review will highlight plant-derived fungicides and chemical constituents that aim to inhibit *P. digitatum* and *P. italicum* in vitro and in vivo. Coatings enriched with plant extracts could be good alternative methods for *Citrus* fruits preservation. Problems and prospects of the research and development of plant-derived natural fungicides will also be discussed in this article.

## 1. Introduction

*Citrus* (Family Rutaceae, *Aurantioideae Citrus*) is considered to be a major fruit crop internationally, with mandarin, orange, pomelo and lemon as prominent varieties. *Citrus* is being cultivated in all parts of the world, but it has a long history spanning 4000 years in the Chinese agriculture system. In China, Guangxi, Jiangxi, Guangdong and Hunan provinces are the major areas of *Citrus* cultivation. Cultivation and selection over the millennia have gradually formed plenty of local varieties including Gannan navel orange, Guangdong sugar orange, Nanfeng tangerine, Wenzhou tangerine and many more. *Citrus* is prone to mechanical damage during picking, packaging, storage, transportation and shelf life. Following skin damage, *Citrus* is vulnerable to a variety of pathogenic fungi. Over 20 different kinds of diseases in *Citrus* during postharvest period have been reported thus far. Among them green and blue molds caused by *P. digitatum* and *P. italicum* respectively, are the worst that easily deteriorate fruits by rotting. The fruit rot rate remains around 10–30% in general, but in severe conditions it increases up to 50% and causes huge economic losses, especially in developing countries [1]. Currently the *Citrus* postharvest diseases are mainly controlled by chemical fungicides such as imazalil, benzimidazole, thiabendazole, prochloraz and pyrimethanil. Many other control methods involving biological and physical treatments are supplemented by using various natural fungicides. Longer and continuous use of chemical fungicides are likely to make the pathogens resistant and pollute the environment. The fungicides that remain on the surface of the *Citrus* peel have also been reported to harm human health. As such, the development of high-efficiency, low-toxic, safe and inexpensive natural plant-derived *Citrus* preservatives are considered of supreme interest.

Rich Chinese flora is reported to possess good antifungal properties and has been widely used in food preservation for centuries [2,3,4]. Huge scientific studies regarding the application of plant extracts in food preservation have reported the potential antifungal agents from this enriched flora [5]. The major sources of *Citrus* antiseptic preservatives have been isolated from members of majority of the plant genus including *Mentha*, *Origanum*, *Thymus*, *Artemisia*, *Heracleum*, *Foeniculum*, *Ligusticum*, *Murraya*, *Phellodendron*, *Cinnamomum*, *Eucalyptus, Amomum, Curcuma*, *Lippia*, *Solanum*, et al. Various advanced techniques have been employed to isolate such antifungal agents, including column chromatography, liquid chromatography, mass spectrometry high performance liquid chromatography (HPLC-MS), gas chromatography-mass spectrometry (GC-MS), and nuclear magnetic resonance spectroscopy (^1^H-NMR, ^13^C-NMR). The studies involving such advanced techniques showed the presence of coumarins, flavonoids, phenolic acids, triterpenoids and natural antibiotics [6,7,8]. The recent trend of studies has been to isolate and screen the active ingredients in the plant extracts. At the same time, the antifungal activity, its mechanism, antifungal properties and structure-activity relationship of active ingredients have not been involved in recent studies. However, it provides a great number of preliminary foundations for the future studies on plant-derived *Citrus* postharvest preservatives. Initially, a sequential screening of postharvest antiseptic preservatives for plant sources was done to isolate hundreds of plant extracts which were found to be effective against *P. digitatum* and *P. italicum*. The current manuscript focused on two major pathogenic fungal strains viz. *P. digitatum* and *P. italicum*, and their inhibition by plant extracts during the past 30 years, application in *Citrus* preservation and antisepsis (Table 1). The manuscript further aims to provide a theoretical basis for the research on the preservative agents for *Citrus* postharvest.

## 2. Studies Regarding the Inhibitory Activity of Plant Extracts on *P. digitatum* and *P. italicum*

### 2.1. Medicinal Plant Extracts

Different medicinal plants have been reported to possess unique uses in the prevention and treatment of diseases in China. Over 5000 different medicinal plants have been reported in Chinese medical system, which mostly bear strong antifungal properties. Herbs and edible plants have a long history of being used as *Citrus* preservatives due to their low toxicity, safety and easy acceptance by consumers. In recent years, many scholars have selected a large number of extracts from herbal medicines that inhibit the activity of *P. digitatum* and *P. italicum.* The effect of rosemary (*Rosmarinus officinalis* L.) extracts on the growth of the green mold of *Citrus* was studied by Hendel et al. [9], and in vitro antifungal assays showed a clear inhibitory effect of the rosemary essential oils and methanol extract on the growth of *P. digitatum*. An aqueous crude extract prepared from lyophilized leaves of *Solanum nigrum* showed an inhibitory effect on *P. digitatum* [10] with a remarkable inhibition zone. *Solanum nigrum* extracts showed an important preventive antifungal effect where 100% of inhibition was observed at 7 days of storage. Previously our lab studied the antifungal effect of ethanolic extracts of *Ficus hirta* Vahl's fruits on *P. italicum* [11], and antifungal assays revealed the presence of a major flavonoid pinocembrin-7-O-β-D-glucoside as a strong antifungal agent. In another study which uses ethyl acetate extracts of *Ramulus cinnamomi* plant showed resistance against several *Citrus* pathogens and good antifungal properties for *P. digitatum* and *P. italicum* [12].

The antifungal activity of medicinal plant extracts is affected by many factors such as origin, season of harvest, processing method and extraction conditions, etc. In order to make extracts that exert optimal antifungal activity, some studies reported the effects of variable extraction conditions on the antifungal activity of plant extracts, and optimized the extraction procedures of some plants with antifungal effects. Our lab [13] has performed certain experiments to optimize the extraction conditions of antifungal extracts from *F. hirta* fruit. The results of this study indicated that the optimal extraction parameters for maximum antifungal activity were: 90% (v/v) ethanol concentration, 65 min extraction time, 31 mL/g solvent to solid ratio at 51 °C. The experimental diameters of inhibition zone (DIZs) values against *P. italicum* and *P. digitatum* were 57.17 ± 0.75 and 39.33 ± 0.82 mm, respectively, which were very close to the values of 57.26 and 39.29 mm predicted by the standard model.

### 2.2. Edible Plants Extracts

Many of the edible plants also have good antifungal effects and can potentially inhibit the growth of postharvest pathogenic fungi in *Citrus* fruits. The extracts of edible plants contain some edible flavorings. Aqueous and 20% ethanolic extracts of garlic (Family Amaryllidaceae) plant showed a significant inhibition of *P. digitatum and P. italicum* [17]. The inhibitory effect of 9 edible wild herbs were studied on common mold of fruits and vegetables, and we found that both *Sanguisorba minor* and *Plantago lanceolata* could significantly inhibit the germination of *P. digitatum* and *P. italicum* spores and the extension of germ tubes [18]. This occurrence is possibly related to the presence of caffeic acid derivatives and flavonoids in extracts. Studies have shown that extracts of soybean flour possess strong antifungal effects [21], and further studies confirmed the various components of soybean protein such as β-conglycinin and glycinin [22]. The results showed that the soy protein fractions contain β-conglycinin significantly inhibited in vitro growth of *P. digitatum* at a wide concentration range (50-3,000 mg/L), and complete inhibition of spore germination at 2,000 mg/L was stated. The soy protein fraction containing β-conglycinin totally inhibited the development of green mold in fruits at 250 mg/L for 7 days after inoculation with the fungal pathogen.

*Aloe vera* (Family Liliaceae) is rich in polysaccharides, proteins, vitamins, active enzymes, lignin, anthraquinones and many other compounds. The anthraquinones are a characteristic component of aloe plant. Moreover, aloe extracts have a wide range of activities to inhibit microbial growth, while aloe saponins and anthraquinones were found as the active ingredients. The extracts were prepared from the aloe gel [23,24], which significantly inhibited the mycelial growth and spore germination of *P. italicum*. The antifungal activity of 8 different aloe species were also examined [25], out of them *A. ferox, A. mitriformis and A. saponaria* showed the highest inhibitory effects on *P. digitatum and P. italicum*, primarily due to the presence of a major component aloin in aloe gel.

Tea saponins are natural non-ionic surfactants isolated from *Camellia sinensis* and are widely used in foods, medicines, pesticides and its emulsifiers. Tea saponins can potentially improve the antifungal activity of *Citrus* chemical fungicides and biocontrol bacteria as well as affecting *Citrus* preservation. A strain of *Bacillus amyloliquefaciens* isolated and identified as HF-01 was found antagonistic to *P. italicum* from the epidermis of seedless granulated oranges [67]. in vitro experiments shown that the inhibitory rate of the antagonistic bacteria against the *P. digitatum* and *P. italicum* were above 96%, the use of HF-01 antagonistic bacteria alone has a certain antiseptic and fresh-keeping effect on the seedless sugar orange, but there is a significant difference compared with the chemical fungicide imazalil. At the same time, 50 μg/mL of tea saponins can significantly improve the fresh-keeping effect of HF-01 antagonistic bacteria (stronger than the activity of the two agents alone), which is not significantly different from chemical fungicides, and has no effect on fruit quality. This indicated that tea saponin and HF-01 have synergistic effects on antagonistic antifungal action. In addition, it was also found that tea saponins have a synergistic effect with other chemical fungicides such as imazalil and prochloraz [26].

### 2.3. Citrus Extracts

**Figure 1:**
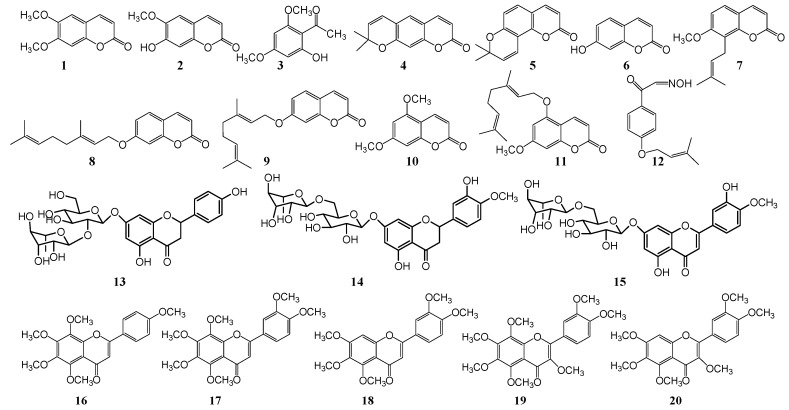
Chemical structures of antifungal constituents 1–20.

*Citrus* itself contains many antifungal substances (Figure 1, Table 2). The presence of essential oils extracted from *Citrus* peel, leaves and flowers by water distillation has strong antifungal effects on both the fungal strains *P. digitatum* and *P. italicum* [51]. Further studies have shown that the *Citrus* peel contains a large number of flavonoids, coumarins and volatile oils, which have the activity to resist the postharvest pathogenic fungi of *Citrus* fruits. *Citrus* peel itself also has a certain ability to resist pathogenic fungi, mainly through endogenous and induced (phytoalexins) antimicrobial substances. *Citrus* coumarins are a class of phytoalexins that inhibit the growth of postharvest pathogenic fungi. Phytoalexins refer to the kind of a substance that inhibits the growth of pathogenic fungi when the pathogenic fungi has invaded the fruits. It means this is the substance that is related to plant resistance to disease. After the *Citrus* fruit is treated by physical (exposure to γ-ray, ultraviolet, heat treatment, skin damage etc.) or chemical (pathogenic fungi, fungicides, etc.) agents, the skin of the *Citrus* fruit can induce some plant antitoxin (mainly coumarins). These phytoalexins are low or absent in normal *Citrus*. When *Citrus* is treated by physical or chemical agents, these phytoalexins can be produced or their presence was significantly increased. These phytoalexins significantly inhibit the growth of pathogenic microorganisms.

Scoparone (1) is the most intensively studied induced plant phytoalexin to date. While it is present in smaller quantities in normal conditions, following exposure to inoculation of pathogens, heat treatment, ultraviolet and γ-rays irradiation etc., its contents significantly increase. The degree of increase is closely related to the conditions of treatment (temperature, energy and time of irradiation), the variety of *Citrus* and the degree of maturity. In general, the content of lemon, kumquat and ponkan is higher, and the content of immature fruit is lower than ripe fruits [68,69,70,71]. Scoparone (1) was first isolated from the grapefruit [53] peel by γ-rays treated. This compound was subsequently isolated from the rind and bark [72,73,74] of other *Citrus* inoculated with *Phytophthora citrophthora.* Its inhibitory activity against *P. digitatum* and *P. italicum* was studied with EC50 of 64 μg/mL and 60 μg/mL respectively. Scoparone (1) and scopoletin (2) were also isolated from the peels of kumquat and *C. sinensis* [59,60], and their content was significantly increased by short-wave ultraviolet irradiation C (UV-C). The peak was reached after 4-10 days of irradiation, and then decreased rapidly, which showed that irradiation can improve the storage stability of kumquat and *C. sinensis*.

In addition to scoparone (1), some other antifungal components of coumarin were isolated and identified from *Citrus*. Two phytoalexins—xanthoxylin (3) and xanthyletin (4) were isolated from the lemon bark [62] and roots [63] inoculated with *P. citrophthora*. 60 μg/mL of xanthyletin (4) can completely inhibit the growth of *P. citrophthora* in *Citrus*. Meanwhile some phenolic components (3,4-dimethoxybenzaldehyde) with xanthyletin (4) can synergistically improve its antifungal activity. Seselin (5) was isolated from the *Citrus* roots [54,75] inoculated with *P. citrophthora*, which is a pyranocoumarin phytoalexin. However, this compound was not detected in other parts, indicating that seselin (5) mainly exists in the roots of *Citrus* trees. Several endogenous coumarins, umbelliferone (6), osthol (7), auraptene (8) and 7-geranoxycoumarin (9), were isolated from the peel of grapefruit [55,56,57], and result showed they have antifungal effects. Angioni et al. [57] further studied the synthetic method of 7-geranoxycoumarin (9), and obtained 7-geranoxycoumarin (9) through the synthesis of geranyl bromide and umbelliferone (6). The inhibitory activities of umbelliferone (6) and 7-geranoxycoumarin (9) against *P. digitatum* and *P. italicum* were compared. The EC50 values were 95 μg/mL and 110 μg/mL, 57 μg/mL and 43 μg/mL, indicating that the structure of umbelliferone (6) can be modified to improve the antifungal activity. Ben-Yehoshua et al. [64,65,66] also found that green-skinned lemons have lower decay rate than yellow-skinned lemons, and their antifungal active substances were studied. One induced and four endogenous antifungal substances in lemon peels were identified by TCL-bioautography, GC-MS and NMR. There are citral, scoparone (1), limettin (10), 5-geranoxy-7-methoxycoumarin (11) and isopimpinellin (32). The EC50 of citral, limettin (10) and 5-geranoxy-7-methoxycoumarin (11) inhibiting the elongation of *P. digitatum* sprouts was 170-242 μg/mL, 860-886 μg/mL and 1578 μg/mL, respectively. The content of limettin (10), which is the active ingredient of green-skinned lemons, is higher than that of yellow-skinned lemons. Therefore, the green-skinned lemon is more resistant to storage. Dubery et al. [76] identified a new inducible antifungal substance, 4-(3-methyl-2-butenoxy) isonitrosoacetophenone (12), from γ-ray treated *Citrus sinensis* and lemon peel.

In addition to coumarin, there are some flavonoids in *Citrus* peels. In particular, polymethoxy- flavones are also resistant to the attack of *P. digitatum and P. italicum*. The waxy components of various *Citrus* peel and hexane extract [61] have resistance effects on a variety of fungi and bacteria, and the MIC concentration of *P. digitatum* is inhibited below 1000 μg/mL. And separating and identifying three activities ingredient: tangeritin (16), nobiletin (17) and scoparone (1). Further studies [77,78] have shown that naringin (13) and hesperidin (14) of dihydrogen flavonoids, diosmin (15) of flavonoids, tangeritin (16), sinensetin (18), heptamethoxyflavone (19) and nobiletin (17) of polymethyl-flavonoids are closely related to the susceptibility of *Citrus* to *P. digitatum*. The high content of flavonoids in *Citrus* peels is resistant to the infection of *Penicillium spp*. Ultraviolet radiation (UV) not only affects the content of coumarin in *Citrus* peel, but also affects the flavonoids. Arcas et al. [79] studied the effect of ultraviolet radiation (UV) on the content of flavonoids in *Citrus aurantium* and the activity of *P. digitatum*. After five days of inoculation with *P. digitatum*, the orange was irradiated by ultraviolet irradiation, and the content of naringin (13) in the peel decreased, while the content of tangeritin (16) increased. The inhibitory activity of tangeritin (16) is stronger than that of naringin (13), which showed that ultraviolet irradiation treatment of *C. aurantium* can improve its antifungal activity.

### 2.4. Volatile Oil

Volatile oil, also known as essential oil, is a volatile oily liquid widely found in plants. It is widely found in Labiatae, Umbelliferae, Lauraceae, Myrtaceae, Rutaceae and Asteraceae families. The peels of the *Citrus* fruit also contain a large amount of volatile oil components.

In addition to flavonoids and coumarins mentioned above, *Citrus* peels also contain volatile oils (Figure 1). The results indicated that *Citrus* essential oils had a good effect on inhibiting the pathogenic fungi of *Citrus* postharvest diseases, which may be related to its natural resistance to pathogens during fruit growth and evolution. The main components of *Citrus* essential oils, including limonene (content over 50% [80]), myrcene, citral, α-pinene, β-pinene, α-terpineol, β-linalool, β-phellandrene and β-ocimene were analyzed by GC-MS. The α-terpineol, β-linalool inhibit the growth of *P. digitatum* and *P. italicum*. However, limonene, myrcene, α-pinene and β-pinene stimulate their growth [58], which may be one of the reasons why damaged *Citrus* are vulnerable to pathogenic fungus. *C. aurantium* and *C. sinensis* volatile oil [52] can inhibit the growth of *Penicillium spp.*, isolated and identified several polymethoxyflavones: tangeritin (16), nobiletin (17), and sinensetin (18), heptamethoxyflavone (19) and quercetogetin (20), *Citrus* polymethoxyflavones can resist a variety of *Citrus* postharvest pathogens (including *P. citrophthora*, *P. italicum* and *Geotrichum*). Caccioni et al. [81] studied the relationship between the volatile oil components of various *Citrus* peels and the inhibition of the activity of *P. digitatum* and *P. italicum*. The antifungal activity of the volatile oils of citrange, lemon, grapefruit and tangerine was strong, while the activity of the volatile oil of sweet orange and sour orange was slightly weak. It was found that the antifungal activity of volatile oil from *Citrus* peel was related to the content of sesquiterpenes and total monoterpenes (except limonene). Sweet orange, sour orange and grapefruit contain more than 90% limonene, and the content of limonene in citrange, lemon and tangerine is about 70%.

**Figure 2:**
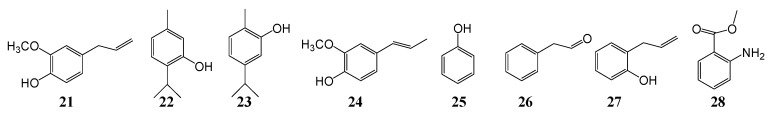
Chemical structures of volatile antifungal constituents 21–28.

In addition to volatile oils of *Citrus* plants, the volatile oils of other plants (Labiatae, Asteraceae and Lauraceae) are also effective at inhibiting *Citrus* postharvest pathogens fungi (Figure 2). The most active ones are volatile oils such as laurel, clove, oregano, cinnamon and mint. CO_2_ supercritical fluid extraction of laurel oil [27] has a good inhibitory effect on postharvest pathogenic fungi, and good results at a concentration of 200 μg/mL. The chemical components of laurel oil were analyzed by GC-MS, and mainly contains 1.8-cineole, linalool, terpineol acetate, methyl eugenol, linalyl acetate, eugenol (21), sabinene, β-pinene and α-terpineol. Yigit et al. [14] studied the inhibitory effect of volatile oils from five plant spices on *P. digitatum* and the preservation effect of *Citrus*. Cumin volatile oil can completely inhibit the growth of mycelium and spore germination. Coriander, rosemary, wild thyme and dill volatile oils also have a certain antifungal effect (mycelial growth inhibition rate is 70–80%). During in vivo experiments, 900 ppm thyme essential oil was found to have a 50% inhibition rate, and other volatile oils had no effect. The results showed that the extracts of essential oil had some differences in fungus inhibition in vitro and in vivo. Hall et al. [16] studied the inhibitory activity of several essential oils on *P. digitatum in vitro*. Results indicated that all essential oils (cinnamon leaf, lemongrass, spearmint, clove leaf and cumin) inhibited the growth (colony and diameter) of both pathogens over untreated PDA plates, but the inhibition was the strongest by clove and cinnamon leaves oil. Tripathi et al. [31] screened the antifungal activity of twenty plants’ volatile oils against *P. italicum*. At the concentration of 500 μg/mL, thirteen plants showed the activity of completely inhibition of mycelium growth. Among them, the IC50 of *Ocimum canum*, wild mint and ginger are 100, 500 and 200 μg/mL, respectively. This is far less than the positive control fungicide benzimidazole (3000 μg/mL). The MIC and MFC concentrations of the peppermint volatile oil [29] against *P. digitatum* were 2.25 mg/mL and 4.5 mg/mL, respectively. Soylu et al. [28] studied the inhibitory effect of volatile oil of five Turkish plants (oregano, fennel, artemisia, laurel and lavender) on *P. digitatum*. Oregano and fennel are most active, the concentrations required to completely inhibit mycelial growth and germ tube elongation was 64 μg/mL and 352 μg/mL, respectively. The other three volatile oils at a concentration 352 μg/mL partially inhibited the elongation of the tube of *P. digitatum*. Zhang et al. [32] when testing for antimicrobial activities of *Melaleuca alternifolia* essential oils showed that mycelial growth and conidial germination were clearly affected by these oils treatments, indicating that these concentrations affected various stages of the development of *P. digitatum* and *P. italicum*. 

Thymol (22) and carvacrol (23) are the two most common volatile components in the Labiatae members (Figure 2). They have good inhibition of plant pathogens activity. These components are mainly distributed in plants such as oregano, thyme, mint and *Elsholtzia*. Yahyazadeh et al. [33] studied the inhibitory effect of volatile oils of four plants (clove, thyme, fennel and sage) on *P. digitatum*. It showed that 600 ppm of clove and thyme volatile oils can completely inhibit the growth of *P. digitatum*. Analysis of their components by using GC-MS indicates that the main components of clove oil are eugenol (21) and β-caryophyllene. The main ingredients of thyme oil are thymol (22), p-cymene and δ-terpinene. Daferera et al. [34] studied the effects of volatile oils from seven Greek aromatic plants on the inhibition of *P. digitatum*, and analyzed the chemical constituents by GC-MC. Four members of the Labiatae including *Origanum vulgare*, *Origanum majorana*, *Origanum dictamus* and *Thymus vulgaris* could completely inhibit the growth of mycelia, spores germinate, and/or form in the concentration range of 250-400 μg/mL. GC-MS analysis of its components found that it mainly contains p-cymene, thymol (22) and carvacrol (23). The IC50 of thymol and carvacrol that inhibiting the growth of *P. italicum* were 47 μg/mL and 79 μg/mL, respectively, and the MIC (minimum inhibitory concentrations) were 160 μg/mL and 200 μg/mL, respectively. The result showed that their antifungal activity has a synergistic effect. Alfonso et al. [82] also studied the inhibition activity of thymol (22) and carvacrol (23) on *P. digitatum* and *P. italicum* by in vitro and in vivo experiments of lemon. The results showed that they significantly inhibited the growth of fungi at a concentration of 250 μg/mL, reduced the rate of decay, exhalation strength, ethylene release and total acid loss of lemon. Scora et al. [83] screened the effects of more than 200 kinds of monoterpenoids and sesquiterpenoids on the inhibition of three *Citrus* postharvest molds. Some phenolic compounds such as eugenol (21), thymol (22), carvacrol (23), isoeugenol (24), phenol (25), phenylacetaldehyde (26), 2-allylphenol (27) and methyl anthranilate (28) were found to have strong inhibitory effects. The volatile oil of *Thymus capitatus* L. [35] can inhibit the growth of *P. digitatum* and *P. italicum* at a concentration of 250 μl/mL. GC-MS analysis mainly contained 81-83% of carvacrol (23). 

### 2.5. Other Plant Extracts

Some studies have also been conducted by scholars in screening other plant extracts that inhibit *P. digitatum* and *P. italicum*. Boubaker et al. [19,20] observed the activity of 71 kinds of Moroccan plant aqueous extracts inhibiting the *P. digitatum* and *P. italicum*, and found that most plants had a certain inhibitory effect, among which 15 plants had an inhibition rate of mycelium growth above 75% under experimental conditions. They were: *Arenaria rubra*, *Anvillea radiata*, *Asteriscus graveolens*, *Bubonium odorum*, *Cistus villosus*, *Halimium umbellatum*, *Hammada scoparia*, *Ighermia pinifolia*, *Inula viscosa*, *Rubus ulmifolius*, *Thymus leptobotrys*, *Peganum harmala*, *Eucalyptus globulus*, *Sanguisorba minor* and *Ceratonia siliqua*. At the same time, 10 mg/mL of *A. graveolens*, *B. odorum* and *H. umbellatum* extracts completely inhibited spore germination, and 1.2 mg/mL of *T. leptobotrys* volatile oil extract completely inhibited the growth of hyphae. They continued to study the antifungal activity of different solvent extracts from eight of the more active plants and found that *A. radiata*, *A. graveolens*, *B. odorum*, *I. viscosa* and *T. leptobotrys* were more active in petroleum ether, chloroform and ethyl acetate extracts. They also found that in the methanol extract of *H. umbellatum*, the petroleum ether extract of *I. pinifolia* and the chloroform extract of *H. scoparia* are highly active. Also, the polarities of the antifungal active substances of different plants are different [36]. Mexican desert plant extracts [38] also inhibit the action of postharvest fungi in fruits. The ethanol extracts of *Lippia graveolens* and *Yucca filifera* Chaub in 1000 μl/L had more than 90% inhibition effect on spore germination of *P. digitatum*, and the growth inhibition rate of mycelium was about 80%. Seven Jordanian plants [84,85] extracts also inhibit the action of *P. digitatum* and *P. italicum*. These were *Fenugreek seeds*, garlic, cinnamon, *Peganum harmala*, *Inula viscosa* and *Solanum nigrum*. Among them, garlic, cinnamon and *Solanum nigrum* methanolic extracts were the most active. Their IC50 is 3.75–18, 5.0–23 and 8.75–24.75 μg respectively. Qasem et al. [15,39] studied the inhibitory effects of some common weed aqueous extracts on three fungi. It was found that six plant extracts including *Crepis aspera*, *Viscum cruciatum*, *Chenopodium murale*, *Sisymbrium irio*, *Solanum nigrum* and *Ranunculus asiaticus* have a good inhibitory effect on *P. digitatum* in *Citrus*. Among them, the *R. asiaticus* can completely inhibit the growth of *P. digitatum* which has been cultured for up to 16 days. H. Boubaker et al. [37] studied the aerial parts of four *Thymus* species in Morocco and found that the main constituents of the extracts of these four plants were carvacrol (76.94%) for *Thymus leptobotrys*, borneol (27.71%) and thymol (18.47%) for *Thymus satureioides* subsp. *pseudomastichina*, camphor (46.17%) and α-terpineol (7.69%) for *Thymus broussonnetii* subsp. *hannonis* and carvacrol (32.24%), γ-terpinene (19.60%) and *p*-cymene (13.52%) for *Thymus riatarum*. The results indicated that *T. leptobotrys* essential oil displayed the highest bioactivity, completely inhibiting the spore germination of *G. citri-aurantii* at 250 μL/L and of *P. digitatum* and *P. italicum* at 500 μL/L. *T. riatarum* essential oil was able to completely inhibit the spore germination of *G. citri-qurantii* (2000 μL/L) and both *Penicillium* species used as target organisms (1000 μL/L). Sayago et al. [40] studied the inhibitory effect of 9 Argentine plant aqueous extracts on two common *Citrus* pathogenic fungi. Three plant extracts of *Chuquiraga atacamensis*, *Parastrephia phyliciformis* and *Parastrephia lepidophylla* have inhibitory effect on *P. digitatum*. The activity is related to the total phenolic content, of which *P. lepidophylla* is the most active, and its MIC and MFC are 400 mg/L and 600 mg/L, respectively. *P. lepidophylla* extract with a total polyphenol content of 600 mg/L and a mixture of fruit wax can reduce the rate of decay in the storage of lemons that have been damaged and inoculated with *P. digitatum*.

### 2.6. Targeted Isolation and Identification of Antifungal Active Ingredients

**Figure 3:**
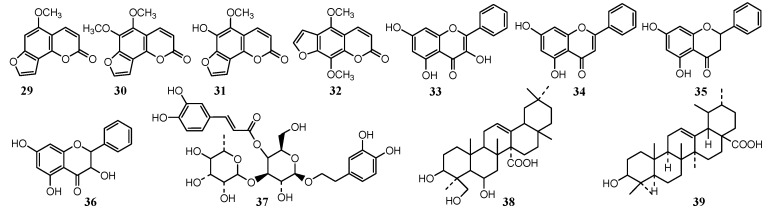
Chemical structures of antifungal constituents 29–39.

The antifungal effect of plant extracts is mainly due to the action of some components or the synergy of several components. It is important to determine the structure of the antifungal components in the plant extracts, while the structure-activity relationship (SAR) and quality control standards of extracts can be further studied. Some scholars have used column chromatography, HPLC-MS and GC-MS to identify active ingredients (Figure 3) in plant extracts with antifungal activity. Li et al. [41] isolated and identified the antifungal active constituents against *Citrus* pathogenic fungi from Radix *Angelicae biseratae*, and identified four coumarin components from the active fraction by GC-MS, namely isobergapten (29) and pimpinellin (30), sphondin (31) and isopimpinellin (32), which are the main antifungal active constituents.

Propolis extract has good antifungal, antiviral, antioxidation, scavenging free radical and anti-tumor activities. Propolis [86] ethyl acetate and ethanol extract can inhibit the spore germination of *P. digitatum* and *P. italicum*, and the mycelium is deformed to inhibit its growth, while the activity is related to the total flavonoid content. Propolis ethanol extract was extracted with systemic solvent, and the antifungal activity showed that ethyl acetate was the most active part. Four flavonoids [87], galangin (33), chrysin (34), pinocembrine (35) and pinobanksin (36), were identified by TCL- bio-autography, column chromatography separation and HPLC-MS technology. The galangin (33) was also identified from the *Helichrysum aureonitens* acetone extract [42] by antifungal activity tracking separation. *P. digitatum* and *P. italicum* were most sensitive to the 0.01 mg/mL concentration of galangin (33), and the inhibition rate is about 30%. Emmanuel et al. [43] studied the activity of two species of *Lippia*, *Lippia javanica* and *Lippia rehmannii*, suppressing a Guazatine-resistant strain of *P. digitatum*. The plant extract of 0.6 mg/mL could inhibit the growth of mycelium, and verbascoside (37) was identified as the main active ingredient.

Gao Kun et al. [45] studied the antifungal active ingredients of *Astilbe myriantha* Diels, and finally isolated and identified an active triterpenoid: 3β, 6β, 24-trihydroxyurs-12-en-27-oic acid (38), whose IC50 was 13.9 mg/L. *Breonadia salicina* acetone extract [46] has an inhibitory effect on variety of *Penicillium*, and the MIC concentration of inhibiting *P. digitatum* is 0.16 mg/mL, from which a major active triterpenoid composition i.e., ursolic acid (39) was isolated and identified.

## 3. Study on the Application of Plant Extracts to *Citrus* Preservation

The effective plant extracts by screening for antifungal activity can be further studied for their application in *Citrus* preservation. Plant extracts can be directly dipped, fumigated, or compounded with other coating materials to improve antifungal and preserved ability. The citral applied as a fumigant was used to study the postharvest diseases of *Citrus* caused by *P. digitatum*, *P. italicum* and *G. citri-aurantii*. The results [88] showed that fumigation of punctured oranges with citral delayed spoilage by sour rot at room temperature by 7–10 days and at 5 °C, by 13–30 days. Meanwhile volatile citral delayed the development of blue mould in abraded, but not punctured, oranges stored at 5 °C. The preserved effect of direct immersion fruit of clove ethanol extract with a good antifungal activity on "Newhall" navel orange was studied previously in our lab [89]. The immersion of fruit of 100 mg/mL clove ethanol extract for 3 min can significantly reduce the rot rate and weight loss of "Newhall", and the rate of decay was only 5.9% after 100 days of storage. However, the decay rate of the control group was 10%. This means that the deal delayed the decrease of TSS, TA and VC content in the pulp, and increased the activity of SOD, POD and chitinase (CHI) during the storage period. Oyourou et al. [44] studied the preserved effect of leaf extracts from two plants (*Lantana camara* and *Lippia javanica*) rich in verbascoside on “valencia” *Citrus*. In the trial, *Citrus* inoculated with *P. italicum* handling with extract containing 1 g/L or 2 g/L. The decay rate of *Citrus* was less than 20% while the control *Citrus* decay rate was 100%. Verbascoside was isolated from *L. camara* and *L. javanica* methanol extracts by column chromatography and high speed countercurrent chromatography (HSCCC). in vitro experiments [30] showed that 500 μl/L of *Lippia scaberrima* and *Mentha spicata* volatile oil, R-(-)-carvone and 1,8-cineole could inhibit *P. digitatum* and the inhibition rate of *P. digitatum* is over 50%. When the concentration reached 3000 μl/L, the inhibition rate was 100%. However, (*d*)-limonene inhibits *P. digitatum* by only 50%. The combination of *L. scaberrima* volatile oil and *Brazilian tropical palm* coating can significantly reduce the rot rate of “Tomango” *Citrus* inoculated with *P. italicum*, and it was not significantly different from chemical fungicides.

Pomegranate peel is the dry peel of pomegranate, which accounts for about 30% of the total weight of pomegranate. It has abroad-spectrum antifungal effect and has obvious inhibitory effects on most Gram-negative bacteria, Gram-positive bacteria, fungi and molds. The fungistatic test in vitro showed that the inhibitory effect of ethanol, methanol and water extracts of pomegranate peel [47] on *P. digitatum* (isolated from grapefruit, lemon and *C. sinensis*) was not significantly different from that of the control with chemical fungicide (imazalil). Further study was conducted on the preservation effect of ethanol extracts of pomegranate peel (0.1, 0.5 and 1.0 g/L) on “Eureka” lemon. The ethanol extracts showed excellent preservation effects by being soaked to the fruit for 3 minutes. The study by Maria G. et al. [48] has also shown that in vitro trials revealed the pomegranate peel extract possess a strong fungicidal activity which against germination of conidia of *Botrytis cinerea*, *P. italicum*, *P. digitatum and P. expansum*. Almost complete inhibition of all fungal spore germination was achieved after 20h of incubation with pomegranate peel extract. At concentrations of 1.2 and 12 g/L complete inhibition of infection was achieved in the majority of host pathogen combinations. Mekbib et al. [49] studied the preservation effect of two Ethiopian plants (*Withania somnifera* and *Acacia seyal*) methanol extract on “Valencia” *Citrus*. They can completely prevent the decay of the damaged fruit and reduce the decay rate of the fruit inoculated *P. digitatum*, which is not significantly different from the chemical fungicide thiabendazole. 

There are diverse application models of plant extracts used in *Citrus* fruits preservation. Other than for direct immersion of the fruits, it can also be compatible with some *Citrus* preservative coating materials or fruit wax to form a composite coating preservative to improve its preservation ability [90]. Chitosan, or chitin, is the product of N-deacetylation chitin. Chitosan is easily dissolved in a weak acid solvent, and the dissolved chitosan solution contains an amino group (NH^2+^), which can inhibit fungi by binding negative electrons. A large number of experimental studies have confirmed that chitosan has a good inhibitory effect on *Citrus* postharvest pathogenic fungi [91]. It is a commonly used *Citrus* postharvest coating material, and it is often compounded with other plant antifungal extracts to improve the preservation performance. The experimental results of our research group [92] showed that the coating significantly reduced the decay rate and weight loss of the navel lorange fruits, delayed the decrease of the content of total soluble solids (TSS), titratable acidity (TA) and vitamin C (Vc), and effectively inhibited the content of MDA. Furthermore, the coatings maintaining enhanced the activity of SOD, CAT, POD and PPO delay the senescence of fruits, and improve the disease resistance of navel orange fruits. Meanwhile, compared with chitosan coating, cinnamaldehyde-chitosani coating could significantly reduce the decay rate and had no adverse effects on fruit quality.

Combined, about 3% of bergamot oil (mainly containing limonene and β-linalool) with chitosan to form composite coating films showed an inhibitory effect on *P. digitatum*. The antifungal effect is better than chitosan alone, and the efficacy corresponds to the content of bergamot oil, where the composite coating effect of 3% bergamot oil has the best result [93]. Inoculation with *P. digitatum Citrus* was treated with two Ethiopian plant extracts (*A. seyal and W. somnifera*) [50]. It can reduce the disease incidence of green mold by 50%, which can also maintain the quality of fruit with the use of fruit wax and reduce weight loss rate. The chemical composition was analyzed by HPLC, and the result found that *A. seyal* mainly contains gallic acid (60.3 mg/mL), while *W. somnifera* contains caffeic acid (8.7 mg /mL), salicylic acid (6.3 mg/mL) and 3, 4-dihydroxybenzoic acid (3.8 mg/mL), indicating that phenolic acids may have antiseptic and preservation properties for *Citrus*. Recent studies by Zambrano-Zaragoza et al. [94] have shown that a variety of nano-composites can be more effective in fungistatic and preservation of coatings, among which Silver nano-particles (AgNPs) with *Fantasia japonica* leaf extract effectively against *E. coli*, *S. aureus* and *P. italicum*. This can be used in *Citrus* fruit preservation, because AgNPs caused cell deformation, cytoplasmic leakage and cell death of *P. italicum*.

## 4. Existing Problems and Future Development Trends

In summary, over the years, many scholars have carried out a large number of experimental explorations on the research of *Citrus* plant-derived fungicides and the development of application dosage forms of *Citrus* botanical fungicides. However, the authors consider that some current problems and future development trends in this field are mainly reflected in the following aspects:

(1) The main pathogen inhibiting activity of *Citrus* were screened out in vitro. The screening of *Citrus* botanical fungicides was based on in vitro. The effects of the fungicides on mycelial growth, spore germination and germ tube elongation of various pathogenic fungi were observed, but the defensive mechanism was not studied in-depth. In addition, the research on *Citrus* preservation is still smaller and only remains at the small-scale experimental stage in the laboratory, and the commercial application of experimental research is even more rare. Plooy et al. [30] studied the effect of coating with various volatile oils (*L. scaberrima*, *Mentha spicata* volatile oils, limonene and R-(-) carvone) on the quality of "Tomango" *Citrus*. The amount of *Citrus* used reached 2.4 tons in the experiment, reaching the commercial application scale.

(2) If the effect is not good, it should be combined with other preservatives or food additives to improve the preservative effect. Compared with chemical fungicides, the antimicrobial and antiseptic effects of plant extracts are still very poor. It is difficult to achieve the ideal preservation effect by using a single plant-derived antiseptic. Several kinds of preservatives with different properties can be used together to improve the antimicrobial effect. For instance, the combination of plant extract tea saponins and prochloraz (chemical fungicide) can improve the activity and reduce the use of prochloraz. Also, the combination of plant volatile oils and other coating materials (fruit wax, volatile oil of *Orbignya martiana* and chitosan) can also improve the antimicrobial effect. Therefore, it is necessary to strengthen the research on compatibility optimization of plant extracts preservatives and other preservative materials.

(3) The antimicrobial and antiseptic effects of *Citrus* plant extracts are closely related to the contents of their active substances. The extraction conditions (extraction solvent, temperature, time and frequency, etc.) are the main factors affecting the contents of the active substances in the plant extracts. In order to make the plant extracts play the best antimicrobial effects for preservation, the optimal extraction process should be further studied by response surface analyses that would illustrate the potential extraction conditions of these plant extracts.

(4) In addition, most of the plant-derived *Citrus* preservatives are plant crude extracts, and the separation and identification of active ingredients is not deep enough. It is difficult to determine the structure of active ingredients, which greatly restricts the research of plant-derived preservatives. Furthermore, the origin, harvesting season, processing methods of plants will affect the content of effective components. Also, the quality of the preservation effect of plant crude extracts is difficult to control. Extraction, isolation, identification of botanical active ingredients form the necessary basis of research and development of botanical *Citrus* preservatives. On the basis of discovering and determining the structure of active compounds, further and systematic studies on structure-activity relationship, pilot optimization, biomimetic synthesis and quality control can be carried out step by step. 

Therefore, in order to develop plant-derived *Citrus* preservatives, it is necessary to further isolate and identify the effective components of plant extracts and study the antifungal mechanisms. Coatings enriched with plant extracts might be good alternative methods for Citrus fruits preservation.

## Figures and Tables

**Table 1:** Inhibitory activity of plant extracts on *P. digitatum* and *P. italicum*.

Plants	Genus	Pathogens	Antifungal Constituents	References
*R. officinalis* L. (rosemary)	*Rosmarinus*	*P. digitatum*	essential oils, methanol extract	[9,14]
*Solanum nigrum*	*Solanum*	*P. digitatum*	aqueous extracts	[10,15]
*Ficus hirta* Vahl’s	*Ficus*	*P. italicum*	pinocembrin-7-O-β-D-glucoside	[11]
*Ramulus cinnamomi*	*Cinnamomum*	*P. digitatum* and *P. italicum*	cinnamaldehyde and cinnamic acid	[12]
*C. zeylanicum*	*Cinnamomum*	*P. digitatum*	volatile oil, eugenol, cinnamaldehyde	[16]
*Allium sativum* (garlic)	*Allium*	*P. digitatum* and *P. italicum*	aqueous and 20% ethanolic extracts, allicin	[17]
*Sanguisorba minor*	*Sanguisorba*	*P. digitatum* and *P. italicum*	derivatives of caffeic acid, apigenin, quercetin, kaempferol	[18,19,20]
*Plantago lanceolata*	*Plantago*	*P. digitatum* and *P. italicum*	caffeic acid derivatives and flavonoids, (iso)verbascoside	[18]
*Glycine max* (soybean)	*Glycine*	*P. digitatum* and *P. glabrum*	β-conglycinin and glycinin	[21,22]
*A. vera*	*Aloe*	*P. digitatum*	aloe saponins and anthraquinones	[23,24]
*A. ferox*	*Aloe*	*P. digitatum and P. italicum*	aloin	[25]
*A. mitriformis*	*Aloe*	*P. digitatum and P. italicum*	aloin	[25]
*A. saponaria*	*Aloe*	*P. digitatum and P. italicum*	aloin	[25]
*C. sinensis*	*Camellia*	*P. digitatum and P. italicum*	tea saponins	[26]
*Laurus nobilis* (laurel)	*Laurus*	*P. digitatum*	volatile oil, contains 1.8-cineole, linalool, terpineol acetate, methyl eugenol, linalyl acetate, eugenol, sabinene, β-pinene, α-terpineol.	[27,28]
*Cuminum cyminum*	*Cuminum*	*P. digitatum*	volatile oil, cuminaldehyde	[14,16]
*Coriandrum sativum*	*Coriandrum*	*P. digitatum*	volatile oil	[14]
*Thymbra spicata* L.	*Thymbra*	*P. digitatum*	volatile oil	[14]
*Anethum graveolens* (dill)	*Anethum*	*P. digitatum*	volatile oil, carvone	[14,16]
*Syzygium aromaticum*	*Syzygium*	*P. digitatum*	volatile oil, eugenol	[16]
*Cymbopogon citratus*	*Cymbopogon*	*P. digitatum*	volatile oil, citral, myrcene	[16]
*Pelargonium graveolens*	*Pelargonium*	*P. digitatum*	volatile oil, citronellol, citronellol formate, gerinol	[16]
*M. piperita* (peppermint)	*Mentha*	*P. digitatum*	volatile oil, menthol, menthone	[16,29]
*M. spicata* (spearmint)	*Mentha*	*P. digitatum*	volatile oil, carvone	[16,30]
*M. arvensis* (wild mint)	*Mentha*	*P. italicum*	volatile oil	[31]
*Ocimum canum*	*Ocimum*	*P. italicum*	volatile oil	[31]
*Zingiber officinale*	*Zingiber*	*P. italicum*	volatile oil	[31]
*Foeniculum vulgare*	*Foeniculum*	*P. digitatum*	volatile oil	[28]
*Artemisia annua*	*Artemisia*	*P. digitatum*	volatile oil	[28]
*Lavandula stoechas*	*Lavandula*	*P. digitatum*	volatile oil	[28]
*Melaleuca alternifolia*	*Melaleuca*	*P. digitatum* and *P. italicum*	essential oil	[32]
*Eugenia caryophyllata*	*Eugenia*	*P. digitatum*	essential oil, eugenol and β-caryophyllene	[33]
*T. vulgaris* (thyme)	*Thymus*	*P. digitatum*	essential oil, thymol, carvacrol, p-cymene and δ-terpinene	[33,34]
*T. capitatus* L.	*Thymus*	*P. digitatum* and *P. italicum*	volatile oil, carvacrol	[35]
*T. leptobotrys*	*Thymus*	*G. citri-aurantii* and of *P. digitatum* and *P. italicum*	volatile oil, petroleum ether, chloroform, ethyl acetate extracts, thymol and carvacrol	[19,20,36,37]
*T. satureioides* subsp. *pseudomastichina*	*Thymus*	*G. citri-aurantii* and of *P. digitatum* and *P. italicum*	borneol and thymol	[37]
*T. broussonnetii* subsp. *hannonis*	*Thymus*	*G. citri-aurantii* and of *P. digitatum* and *P. italicum*	camphor and α-terpineol	[37]
*T. riatarum*	*Thymus*	*G. citri-aurantii* and of *P. digitatum* and *P. italicum*	carvacrol, γ-terpinene and *p*-cymene	[37]
*O. syriacum*	*Origanum*	*P. digitatum*	volatile oil	[28]
*O. vulgare*	*Origanum*	*P. digitatum*	essential oil, thymol and carvacrol	[34]
*O. majorana*	*Origanum*	*P. digitatum*	essential oil, thymol and carvacrol	[34]
*O. dictamus*	*Origanum*	*P. digitatum*	essential oil, thymol and carvacrol	[34]
*Arenaria rubra*	*Arenaria*	*P. italicum*	aqueous extract	[19,20]
*Anvillea radiata*	*Anvillea*	*P. italicum*	petroleum ether, chloroform, ethyl acetate extracts	[19,20,36]
*Asteriscus graveolens*	*Asteriscus*	*P. italicum*	petroleum ether, chloroform, ethyl acetate extracts	[19,20,36]
*Bubonium odorum*	*Bubonium*	*P. italicum*	petroleum ether, chloroform, ethyl acetate extracts	[19,20,36]
*Cistus villosus*	*Cistus*	*P. digitatum* and *P. italicum*	aqueous extract	[19,20]
*Halimium umbellatum*	*Halimium*	*P. italicum*	methanol extract	[19,20,36]
*Hammada scoparia*	*Hammada*	*P. italicum*	chloroform extract	[19,20,36]
*Ighermia pinifolia*	*Ighermia*	*P. italicum*	petroleum ether extract	[19,20,36]
*Inula viscosa*	*Inula*	*P. italicum*	petroleum ether, chloroform, ethyl acetate extracts	[19,20,36]
*Rubus ulmifolius*	*Rubus*	*P. italicum*	aqueous extract	[19,20]
*Pegan* *um harmala*	*Pegan* *um*	*P. digitatum* and *P. italicum*	aqueous extract	[19,20]
*Eucalyptus globulus*	*Eucalyptus*	*P. digitatum* and *P. italicum*	aqueous extract	[19,20]
*Ceratonia siliqua*	*Ceratonia*	*P. digitatum*	aqueous extract	[19,20]
*Yucca filifera* Chaub	*Yucca*	*P. italicum*	ethanolic and hexanic extracts	[38]
*Chenopodium murale*	*Chenopodium*	*P. digitatum*	aqueous extract	[15,39]
*Crepis aspera*	*Crepis*	*P. digitatum*	aqueous extract	[15,39]
*Ranunculus asiaticus*	*Ranunculus*	*P. digitatum*	aqueous extract	[15,39]
*Sisymbrium irio*	*Sisymbrium*	*P. digitatum*	aqueous extract	[15]
*Chuquiraga atacamensis*	*Chuquiraga*	*P. digitatum*	aqueous extracts, total phenolics	[40]
*Parastrephia phyliciformis*	*Parastrephia*	*P. digitatum*	aqueous extracts, total phenolics	[40]
*Parastrephia lepidophylla*	*Parastrephia*	*P. digitatum*	aqueous extracts, total phenolics	[40]
*Angelicae biseratae*	*Angelicae*	*P. digitatum* and *P. italicum*	Coumarins, isobergapten, pimpinellin, sphondin, isopimpinellin	[41]
*Helichrysum aureonitens*	*Helichrysum*	*P. digitatum* and *P. italicum*	galangin	[42]
*L. graveolens*	*Lippia*	*P. digitatum*	ethanolic and hexanic extracts	[38]
*L. javanica*	*Lippia*	*P. digitatum* and *P. italicum*	verbascoside	[43,44]
*L. rehmannii*	*Lippia*	*P. digitatum*	verbascoside	[43]
*L. scaberrima*	*Lippia*	*P. digitatum*	volatile oil, (*d*)-cinene, R-(-)-carvone and 1,8-cineole	[30]
*Astilbe myriantha* Diels	*Astilbe*	*P. digitatum*	triterpenoid, 3β, 6β, 24-trihydroxyurs-12-en-27-oic acid	[45]
*Breonadia salicina*	*Breonadia*	*P. digitatum*	triterpenoid, ursolic acid	[46]
*Lantana camara*	*Lantana*	*P. italicum*	verbascoside	[44]
*Punica granatum*	*Punica*	*P. italicum* and *P. digitatum*	ethanol, methanol and water extracts	[47,48]
*Withania somnifera*	*Withania*	*P. digitatum*	caffeic acid, salicylic acid, 3, 4-dihydroxybenzoic acid	[49,50]
*Acacia seyal*	*Acacia*	*P. digitatum*	methanol extracts, gallic acid	[49,50]

**Table 2:** Inhibitory activity of *Citrus* extracts on *P. digitatum* and *P. italicum.*

*Citrus* Plants	Other name	Pathogens	Antifungal Constituents	References
*C. aurantium*	sour orange	*P. citrophthora*, *P. italicum* and *Geotrichum*	essential oils, polymethoxyflavones, tangeritin, nobiletin, sinensetin, heptamethoxyflavone and quercetogetin	[51,52]
*C. paradisi* L.	grapefruit	*P. digitatum* and *P. italicum*	coumarins including Scoparone, seselin, umbelliferone, osthol, auraptene 7-geranoxycoumarin; essential oils, limonene, α-pinene, sabinene, myrcene, α-terpineol, linalool, citral, nootkatone	[53,54,55,56,57,58]
*C. japonica*	kumquat	*P. digitatum*	scoparone and scopoletin	[59]
*C. sinensis*	sweet orange	*P. citrophthora*, *P. italicum* and *Geotrichum*	scoparone and scopoletin, volatile oil, limonene, α-pinene, sabinene, myrcene, α-terpineol, linalool, citral; polymethoxyflavones	[52,58,60,61]
*C. limon*	lemon	*P. citrophthora*, *P. digitatum* and *P. italicum*	waxy components, hexane extract, scoparone, xanthoxylin and xanthyletin; limettin, isopimpinellin, 5-geranoxy-7-methoxycoumarin and Scoparone; volatile oil, citral	[61,62,63,64,65,66]
*C. reticulata*	mandarin	*P. digitatum*	waxy components, hexane extract, tangeritin, nobiletin	[61]
*C. clementina*	clementine	*P. citrophthora*, *P. italicum* and *Geotrichum*	volatile oil, nobiletin, and sinensetin, heptamethoxyflavone, limonene, α-pinene, sabinene, myrcene, α-terpineol, linalool	[52,58]
